# Personal protective equipment availability and accessibility among nurses and midwives in selected urban general hospitals in Lusaka, Zambia: a cross-sectional study

**DOI:** 10.11604/pamj.2023.44.52.32936

**Published:** 2023-01-25

**Authors:** Sebean Mayimbo, Kabwe Chitundu, Samson Shumba, Nedah Chikonde Musonda, Mutinke Zulu, Deborah Nayame Mushamba, Lonia Mwape, Patricia Katowa-Mukwato

**Affiliations:** 1 Department of Midwifery, Women and Child Health, School of Nursing Sciences, University of Zambia, Lusaka, Zambia,; 2 Department of Mental Health and Psychiatry Nursing, School of Nursing Sciences, University of Zambia, Lusaka, Zambia,; 3 Department of Epidemiology and Biostatistics, School of Public Health, University of Zambia, Lusaka, Zambia,; 4 Ministry of Health, Ndeke House, Lusaka, Zambia,; 5 Women and Newborn Hospital, University Teaching Hospitals, Lusaka, Zambia,; 6 Mental Health Nursing Department, School of Medicine, Levy Mwanawasa Medical University, Lusaka, Zambia,; 7 Department of Basic and Clinical Sciences, School of Nursing Sciences, University of Zambia, Lusaka, Zambia

**Keywords:** Availability, accessibility, personal protective equipment, COVID-19, nurses, midwives, gowns, masks, pandemic, antenatal care

## Abstract

**Introduction:**

the World Health Organization (WHO) declared COVID-19 a pandemic in January 2020, which has spread to many countries, including Zambia. Zambia has had challenges in providing personal protective equipment (PPEs) to nurses and midwives. The study's objective was to assess the availability and accessibility of PPEs among nurses and midwives caring for women in the general hospitals in Lusaka, Zambia.

**Methods:**

a cross-sectional analytical study design was conducted at five general hospitals in Lusaka on 162 nurses and midwives between February and April 2021, selected by purposive sampling of study sites and simple random sampling to select the participants. Data was collected using a semi-structured self-administered questionnaire and analyzed in STATA version 13. Chi-square and Fisher's exact test were used to test associations between the independent variables and the outcome, and a multivariable logistic regression was used to investigate the predictors of accessing PPEs.

**Results:**

out of the 162 who participated in the study, 48.8% were nurses, while 51.2% were midwives. Only 10% (16/160) of the participants reported having enough PPEs at work. Age, marital status, PPE use, employment duration, and protection confidence were associated with accessibility (P<0.05).

**Conclusion:**

overall, there was an inadequate provision of PPEs in the health facilities putting the nurses and midwives at a high risk of acquiring COVID-19. Policymakers need a deliberate move to make the availability and accessibility of PPEs a reality during the pandemic.

## Introduction

COVID-19, or severe acute respiratory syndrome coronavirus 2, is an infectious respiratory disease caused by a novel coronavirus (SARS-CoV-2) [1-3]. COVID-19 emerged from China in Wuhan City, Hubei province, in 2019 [4-6]. Since then, the virus has spread to almost all parts of the world, including Zambia [7,8]. COVID-19 is spread by droplets from person to person [9]. When an infected individual coughs or sneezes, droplets from the infected person's cough or sneeze enter the mouth or nostrils of someone within close proximity, it can also be caught by touching infected hard surfaces and then touching the mouth, nose, and eyes with the same hands [9].

In order to protect the health care providers from being infected whilst attending to patients suffering from COVID-19, it is necessary that they wear PPEs [10-12]. Personal protection equipment is an attire that protects the user from work-related health and safety hazards. Personal Protective equipment comes in many forms, such as surgical masks, non-surgical masks, gloves, goggles, face shields, gowns and N95 masks [13]. In many countries around the world, COVID-19 has resulted in a significant shortage of PPEs), putting a strain on medical services during this crisis [14-17].

Concerns about a sufficient supply of PPEs, as well as the shifting nature of the current epidemic, which has many personnel operating in unfamiliar places with unfamiliar equipment, could exacerbate concerns about inappropriate PPE use and the risks it poses (UK, 2020). Previous studies have shown that high-quality PPE is an effective and efficient means of keeping health care workers safe [18]. Many countries, including the United States of America, have reported a severe shortage of PPEs at one time or another during the pandemic [19-21]. This was exacerbated by an increase in demand which triggered the consequences of a lack of PPEs. Inadequate PPEs increase the likelihood of infection to COVID-19 by health care providers [22]. The situation has not been any different from what is happening in Zambia [23-25].

Nurses and midwives are at the helm of care during the Pandemic and might be at higher risk of contracting COVID-19 [26] from patients compared to any other categories of staff [27]. This study, therefore, was aimed at assessing the availability and accessibility of COVID-19 PPEs among nurses and midwives in selected urban general hospitals in Lusaka, Zambia. Methods used in the data, results, discussion and conclusion are included in the study.

## Methods

**Study design, setting, and population:** this was an analytical cross-sectional study conducted between December 2020 and May 2021 on a sample of 162 nurses (79) and midwives (83). The study was conducted at the five general hospitals in the Lusaka Urban District, namely Chilenje, Kanyama, Chipata, Matero, and Chawama. The selected health facilities and participants were chosen purposefully because they cater to clients at first contact before referring them to the tertiary facility, rendering them at risk of receiving and caring for women who may be asymptomatic or symptomatic but undiagnosed for COVID-19. The general hospitals all have a Maternal and Child Health Department, a labour ward, and antenatal and postnatal wards. In addition, they all have qualified obstetricians. All nurses and midwives who had worked for two weeks or more and consented were recruited to the study, whereas those who had worked for less than two weeks were excluded because they had not yet familiarized themselves with the ward routines.

**Sample size and sampling technique:** to achieve a minimum power of 80%, the study used a proportion of 10% availability of Personal Protective Equipment´s (PPEs) and non-response set at 10% (to account for missing data) to give a minimum required sample size of 154. Participants were randomly sampled from each health facility to give a total number of 162 participants in the study. Five health facilities were purposively selected (Chilenje, Matero, Kanyama, Chawama and Chipata) and participants were selected at random between February and April of 2021.

**Data collection tool:** the purpose of the study was explained to the participants. Consenting nurses and midwives were added to a WhatsApp group and provided a link to an online survey monkey as utilized by other researchers [28,29]. A self-administered questionnaire was administered to the participants and had three sections; questions on socio-demographic details, questions on the availability of PPEs, and third on the accessibility of PPEs.

**Variables:** the outcome variable was personal protective equipment, whereas the independent variables were socio-demographic characteristics such as sex, professional qualifications, the name of the hospital, and the number of years in service. The others were the type of PPEs such as surgical masks, eye goggles, and gloves; the availability of PPEs such as questions if the PPEs were reused and if the members of staff were able to fit into the protective clothing. The other variable was the accessibility of the PPEs.

**Data analysis:** in the descriptive statistics, frequencies and percentages were computed for categorical and dichotomous variables. Age (continuous variable) was checked for normality using the Shapiro-Wilk test, and reporting was done using the median and interquartile range (IQR) since data was not normally distributed. A Kruskal-Wallis or ranksum test was used to measure the association of age and accessibility to standard PPEs (gowns, gloves, surgical mask fit tested N95 or FFP2 respirator, and eye protection (goggles or face shield) [30]. Furthermore, to determine the association between categorical variables, a Chi-square or Fisher's exact test was used (if the expected values in the contingency cell were less than five, a Fisher's exact test was used; otherwise, a Chi-square test was preferred). Logistic regression was used to determine the predictors of accessibility to PPEs. The likelihood ratio test, Akaike's Information Criteria (AIC), and Bayesian Information Criteria (BIC) were used to come up with the best model. A P-value of < 0.05 was considered significant.

**Ethical consideration:** this study was approved by the University of Zambia Biomedical Research Ethics Committee (UNZABREC-ref: 1083-2020) and the National Health Research Authority (NHRA). Consent was obtained from the participants before undertaking the study. Only nurses and midwives who gave consent were included in the study. Utilizing special codes to identify participants allowed for the preservation of anonymity and privacy. Participation was voluntary, and no incentives were provided to the respondents.

## Results

**Participants' socio-demographic characteristics concerning the availability of PPEs:** the overall population of the data showed that the median age was 30 years (IQR 26-36). Of the population, 87.65% (142/162) were females, and 12.35% (20/162) were males. The majority of the respondents came from medium-density areas, accounting for 72.84% (118/162), high-density areas were 15.43% (25/162), and low-density areas were 11.78% (19/162). The overall population had 51.23% (83/162) midwives and 48.77% (79/162) nurses. The majority of those, 39.66% (23/58), were from Kanyama general hospital, while the least, 1.72% (1/58), were from Matero general hospital. Among the respondents, most of the nurses and midwives were from the Labour Ward Department, 45.96% (74/161) and the least were from postnatal wards, 9.94% (16/161). The results showed that 45.68% (74/162) of the respondents had worked for one to five years.

In the current study, 97.53% (158/162) reported that surgical masks were available, 0.62% (1/162) had eye goggles, and 93.21% (151/162) had gloves. On other specific PPEs, only 3.70% (6/162) and 97.53% (158/162) responded that long-sleeved gowns and respiratory masks were available, respectively. Two-thirds, 67.28% (109/162), confirmed the availability of aprons in the study. 75.78% (122/162) of the respondents claimed that they lacked sufficient PPEs. More than half of the population (58.64%) reused PPEs in different health facilities, and about 80% (128/160) agreed to have access to the PPEs.

**The availability of PPEs and socio-demographic characteristics of health practitioners**: Table 1 below shows the baseline characteristics of the availability of PPEs among the study population. More nurses (57 (46.72%)) and midwives (65 (53.28%)) reported not having enough PPEs available for them. Similarly, most of the nurses and midwives in the labour ward, ANC, postnatal and other departments reported not having enough PPEs (63 (52.07%), 21 (17.36%), 12 (9.92%), and 25 (20.66%), respectively). The results of the study show that 96.72% (118/122) who had surgical masks reported not having enough of them, only 1 (0.82 %) had an eye goggle, and 121 (99.18%) who did not have reported that they were not enough. A more significant proportion of nurses and midwives who had gloves and aprons (92.62% and 65.57%, respectively) reported that they were insufficient. However, a more significant proportion did not have long-sleeved gowns (96.72%) and respiratory masks (97.54%) and reported that they were insufficient.

**Table 1:** baseline characteristics of availability of personal protective equipment

Characteristics	Availability of PPEs				P-value
	Enough at work place	Enough but worried	Not enough	Don't know	
**Qualification**					
Nurse	10 (62.50)	9 (47.37)	57 (46.72)	2 (50.00)	0.711^C^
Midwife	6 (37.50)	10 (52.63)	65 (53.28)	2 (50.00)	
**Hospital**					
Chilenje	1 (33.33)	0 (0.00)	16 (32.00)	0 (0.00)	0.473^F^
Matero	0 (0.00)	0 (0.00)	1 (2.00)	0 (0.00)	
Kanyama	2 (66.67)	3 (75.00)	18 (36.00)	0 (0.00)	
Chawama	0 (0.00)	1 (25.00)	5 (10.00)	0 (0.00)	
Chipata	0 (0.00)	0 (0.00)	10 (20.00)	1 (100.00)	
**Department**					
Labour ward	3 (18.75)	7 (36.84)	63 (52.07)	1 (25.00)	0.044^F^
Antenatal	9 (56.25)	4 (21.05)	21 (17.36)	1 (25.00)	
Postnatal	1 (6.25)	3 (15.79)	12 (9.92)	0 (0.00)	
Others	3 (18.75)	5 (26.32)	25 (20.66)	2 (50.00)	
**Duration in employment**					
Less than 1 year	3 (18.75)	4 (21.05)	17 (13.93)	1 (25.00)	0.903^C^
1 to 5 years	8 (50.00)	8 (42.11)	56 (45.90)	1 (25.00)	
Greater than 5 years	5 (31.25)	7 (36.84)	49 (40.16)	2 (50.00)	
**Reusing PPEs**					
No	16 (100.00)	12 (63.16)	65 (53.72)	2 (50.00)	
Yes	0 (0.00)	7 (36.84)	56 (46.28)	2 (50.00)	0.005^F^
Accessible					
No	11 (68.75)	13 (68.42)	99 (82.50)	4 (100.00)	
Yes	5 (31.25)	6 (31.58)	21 (17.50)	0 (0.00)	0.233^F^
**Confidence of protection from PPEs**					
Very confident	1 (6.25)	2 (10.53)	2 (1.65)	0 (0.00)	
Moderately confident	10 (62.50)	7 (36.84)	21 (17.36)	0 (0.00)	
Slightly confident	5 (31.25)	4 (21.05)	35 (28.93)	3 (75.00)	
Not confident at all	0 (0.00)	6 (31.58)	63 (52.07)	1 (25.00)	<0.0001^F^
**Able to fit in PPE**					
No	4 (25.00)	12 (63.16)	82 (69.49)	3 (75.00)	
Yes	12 (75.00)	7 (36.84)	36 (30.51)	1 (25.00)	0.005^F^

C=Chi-square test; F= Fishers exact test; K = Kruskal Wallis test

The results further showed that there was not enough availability of PPEs was the most predominant response among both those that either reused PPEs or those who did not (65 (53.72%) and 56 (46.28%), respectively). Similarly, most respondents who felt very confident 2 (10.53%), moderately 21 (17.36%), slightly 35 (28.93%) and not at all confident 63 (25.07%) still reported not enough availability of PPEs. There were high numbers of nurses and midwives who were not able to fit 82 (69.49%), and those who were able to fit 36 (30.51%) in the available PPEs reported as well that there were not enough PPEs available. In the current study, only feeling protected, being able to fit in, and the department where the nurses and midwives were working were associated with the availability of PPEs (P<0.05).

**Accessibility to PPEs and socio-demographic characteristics of health practitioners:** the results in Table 2 below show the baseline characteristics of accessibility of PPEs and socio-demographic factors among the health practitioners. The results show that the median age for those that did not have access to the PPEs was 32 (IQR, 27-38) years old, and 27 (IQR, 25-31) years old for those that had access. The majority of nurses and midwives who did not access the PPEs belonged to labour wards 57/128 (44.53%). Accessibility to PPEs was 65.63% for those who had enough PPEs, but 18.75% and 25.63% for those who had enough but were worried and those who had enough at work, respectively.

**Table 2:** baseline characteristics of accessibility of PPEs

Characteristics	Accessibility of PPEs		P-value
	No	Yes	
**Age, median (IQR) years**	32 (27 -38)	27 (25ï¿½31)	0.008^R^
**Sex**			0.232^C^
Male	14 (10.94)	6 (18.75)	
Female	114 (89.06)	26 (81.25)	
**Qualification**			0.812^C^
Nurse	61 (47.66)	16 (50.00)	
Midwife	67 (52.34)	16 (50.00)	
**Hospital**			0.779^F^
Chilenje	12 (25.53)	3 (33.33)	
Matero	1 (2.13)	0 (0.00)	
Kanyama	18 (38.30)	5 (55.56)	
Chawama	6 (12.77)	0 (0.00)	
Chipata	10 (21.28)	1 (11.11)	
**Department**			0.421^F^
Labour ward	57 (44.53)	17 (53.13)	
Antenatal	26 (20.31)	8 (25.00)	
Postnatal	15 (11.72)	1 (3.13)	
Others	30 (23.44)	6 (18.75)	
**Duration in employment**			0.017^C^
Less than 1 year	18 (14.06)	7 (22.58)	
1 to 5 years	54 (42.19)	19 (61.29)	
Greater than 5 years	56 (43.75)	5 (16.13)	
**Surgical masks**			0.585^F^
Not available	4 (3.13)	0 (0.00)	
Available	124 (96.88)	32 (100.00)	
**Eye goggles**			0.200^F^
Not available	128 (100.00)	31 (96.88)	
Available	0 (0.00)	1 (3.13)	
**Gloves**			1.000^C^
Not available	9 (7.03)	2 (6.25)	
Available	119 (92.97)	30 (93.75)	
**Long sleeved gowns**			0.345^F^
Not available	124 (96.88)	20 (93.75)	
Available	4 (3.13)	2 (6.25)	
**Respiratory masks**			
Not available	125 (97.66)	31 (96.88)	
Available	3 (2.34)	1 (3.13)	0.800^F^
**Apron**			
Not available	44 (34.38)	9 (28.13)	
Available	84 (65.63)	23 (71.88)	0.502^C^
**Reuse**			
No	60 (46.88)	26 (83.8)	
Yes	68 (53.13)	5 (16.13)	0.002^C^
**Availability of PPEs**			
Enough at workplace	11 (8.66)	5 (15.63)	
Enough but worried	13 (10.24)	6 (18.75)	
Not enough	99 (77.95)	20 (65.63)	
Don’t know	4 (3.15)	0 (0.00)	0.233^F^
**Feel protected**			
Very confident	3 (2.34)	2 (6.45)	
Moderately confident	26 (20.31)	12 (38.71)	
Slightly	38 (29.69)	9 (29.03)	
Not confident at all	61 (47.66)	8 (25.81)	0.041^F^
**Able to fit**			
No	80 (64.00)	20 (64.52)	
Yes	45 (36.00)	11 (35.48)	0.957^C^
**Appropriate**			
No	39 (30.71)	12 (37.50)	
Yes	88 (69.29)	20 (62.50)	0.462^C^

R = Ranksum; C = Chi-square test F= Fisher exact test

The findings also show that most respondents who did not reuse the PPEs (53.13%) were not accessing them compared to those who reused them (46.88%). The ones who felt moderately confident (38.71%), slightly confident (29.03%), not confident at all (25.81%), and very confident (6.45%) in protection responded yes to accessibility. However, the study showed that only age, marital status, reusing PPEs, duration of employment, and feeling of being protected from infection were associated with accessibility (P<0.05).

**Univariate and multivariable logistic regression model:** the results in Table 3 below show the univariate and multivariable logistic regression model of accessibility to PPEs. Controlling for other factors, a year increase in the age of a health practitioner (nurse or midwife) reduced the odds of accessing PPEs by a factor of 0.95 times (95% CI, 0.86-1.04), but there was no sufficient evidence to suggest an association. Similarly, males compared to females had reduced odds of accessing PPEs (AOR, 0.56; 95% CI, 0.14-7.47; P = 0.430), albeit the effect was also not statistically significant.

**Table 3:** the univariate and multivariable logistic regression model

Variables	OR (95%CI)	P-value	AOR (95% CI)	P-value
Age	0.92 (0.86 – 0.98)	0.010	0.95 (0.86 – 1.04)	0.277
**Sex**				
Males	Ref (1)		Ref (1)	
Females	0.64 (0.21 – 1.93)	0.427	0.56 (0.14 – 2.34)	0.430
**Department**				
Labour ward	Ref (1)		Ref (1)	
Antenatal	0.90 (0.33 – 2.44)	0.840	1.73 (0.40 – 7.47)	0.461
Postnatal	0.22 (0.03 – 1.82)	0.161	0.13 (0.01 – 1.36)	0.088
Others	0.67 (0.24 – 1.88)	0.447	0.76 (0.21 – 2.84)	0.687
**Duration of employment**				
Less than 1 year	Ref (1)		Ref (1)	
1 to 5 years	1.06 (0.37 – 3.05)	0.920	1.56 (0.42 – 5.72)	0.505
Greater than 5 years	0.27 (0.07 – 0.98)	0.047	0.51 (0.09 – 2.74)	0.431
**Feel protected**				
Very confident	Ref (1)			
Moderately	0.69 (0.10 – 4.70)	0.707	1.00 (0.09 – 11.2)	0.998
Slightly	0.36 (0.05 – 2.45)	0.294	0.42 (0.03 – 5.00)	0.490
Not confident at all	0.20 (0.03 – 1.36)	0.100	0.35 (0.03 – 3.92)	0.393
**Able to fit in PPEs**				
No	Ref (1)		Ref (1)	
Yes	1.03 (0.45 – 2.35)	0.946	0.88 (0.29 – 2.68)	0.824
**Reuse of PPEs**				
No	Ref (1)		Ref (1)	
Yes	0.23 (0.08 – 0.63)	0.004	0.16 (0.04 – 0.58)	0.005
**Availability of PPEs**				
Enough at workplace	Ref (1)		Ref (1)	
Enough but worried	1.02 (0.24 – 4.26)	0.983	3.61 (0.55 – 23.77)	0.181
Not enough	0.44 (0.39 – 1.42)	0.171	1.78 (0.32 – 9.94)	0.512
Don’t know	-	-	-	-
**Access to Appropriate PPEs**				
No	Ref (1)		Ref (1)	
Yes	0.81 (0.35 – 1.84)	0.609	0.62 (0.22 – 1.77)	0.371

Reusing PPEs by health practitioners reduced the odds of accessing PPEs by a factor of 0.16 times compared to those who never reused (95% CI, 0.04-058; P = 0.005), and this effect was statistically significant. On the other hand, the ANC departments had increased odds of accessing PPEs compared to the labour wards (AOR, 1.73; 95% CI, 0.40-7.47; P=0.461). However, staff in postnatal and other departments had reduced odds of accessing PPEs compared to the labor ward department (AOR, 0.13; 95% CI, 0.01 - 1.36; P =0.088) and (AOR, 0.76; 95% CI, 0.21 - 2.84; P =0.687) respectively. The results show that confidence in the use of PPEs, duration of employment of the health practitioners, availability of PPEs, and access to appropriate PPEs were not predictive of accessibility to standard PPEs (P ≥ 0.05).

The univariate model showed that the age and duration of employment of health practitioners were statistically significant (P < 0.05). However, in the adjusted model, they were both insignificant (P ≥ 0.05). The results were first run in the mixed effect logistic regression, and the variance found was zero, which suggested that we did not have to worry about intra-cluster variance. The best model was selected by AIC and BIC. The likelihood ratio test from the best-fit model also suggested that this model was better than the null model (P = 0.002).

**Figure 1:**
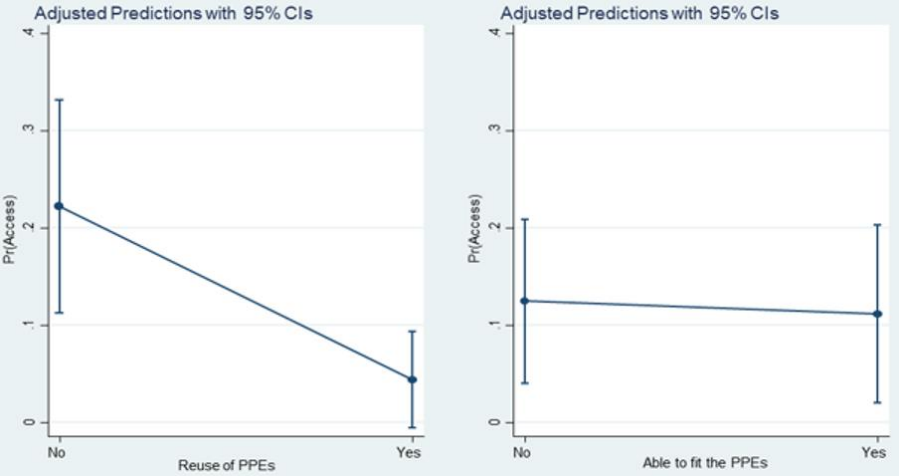
margin plots for reuse and fitting of PPEs among health practitioners

**Predictive margins:** when margins plots were explored, the findings showed that those who did not reuse PPEs had a higher probability of accessing PPEs compared to those who reused them. Similarly, the health practitioners who reported being unable to fit into the PPEs had a relatively higher probability of accessing PPEs compared to those who were able to fit into the PPEs, albeit the difference was relatively small. However, for the reuse of PPEs, only the probability for no was significantly different from zero (p < 0.0001), while the margin probabilities were significantly different from zero for both the yes and no responses (P = 0.004 and P = 0.018, respectively) (Figure 1).

## Discussion

**Socio-demographic characteristics of the participants:** the median age of nurses and midwives in this study of 30 years is similar to a study conducted in Turkey, where the mean age was 30.5 [31]. The above findings might represent the average age at which an individual is expected to have completed school and college in both countries. The high number of females of 87.65% versus 12.35% males in our study reveals that the nursing career is predominantly female [32]. The higher number of midwives compared to nurses in our study is because the study sites were mainly maternity wings where most staff were midwives.

**Availability and accessibility of PPEs:** as much as most of the nurses and midwives reported having access to surgical masks, with a reported availability of only 97% in our study, other essential PPEs such as gowns and goggles were not readily available, showing that the health workers were susceptible to COVID-19 infection. For example, although only one person had eye goggles, it exacerbated the chances of infection as COVID-19 can spread via the epithelium of the conjunctiva [33,34]. Approximately 75% of the nurses and midwives reported not having adequate PPEs, similar to a study conducted in China [35] and another in Afghanistan [36], although the percentages were not reported. The non-availability of gloves, surgical masks, and respirators in these settings is contrary to the guidelines by the World Health Organization and puts the nurses and midwives at a high risk of contracting COVID-19 [37]. One of the predisposing factors to the shortage of PPEs during the COVID-19 Pandemic is the reduction in the global supply chain [38]. The inadequate availability and accessibility of PPEs in our study are similar to an international survey by Tabah and colleagues, who equally reported similar findings [39].

The fact that the labour ward was reported to have more shortages of PPEs than other departments might be due to the high numbers of patients [40,41]. Due to many patients, most nurses and midwives reported reusing PPEs, which is not ideal for preventing the spread of COVID-19 [42], but this was unavoidable during the Pandemic and happened even in developed countries [42,43]. COVID-19 harmed most nations in the world, as evidenced by the fact that conditions were similar in most of the settings [44]. An international survey conducted among healthcare workers working in intensive care units was conducted to assess the availability and use of PPEs. The results equally revealed similar outcomes [39] in keeping with our study. Additionally, Wakgari and colleagues (2021) conducted a study in Ethiopia revealing that gloves and gowns were the most frequently unavailable PPEs, somewhat similar to ours [45]. Another study revealed the unavailability of gloves in hospitals in England during the COVID-19 Pandemic [46].

The implication of the inadequate availability and inaccessibility of PPEs during COVID-19 is that many healthcare personnel are unsure how to fulfil their medical duties safely and effectively under these difficult conditions [22,47]. Frontline health care workers were reported to have continued caring for COVID-19 patients despite many problems such as inadequate PPEs, insufficient training, and inconsistent supervision [48]. Our study, however, did not examine how nurses and midwives handled client care when PPEs were either scarce or nonexistent. Caring for clients when PPEs are in short supply is challenging to measure as health care providers might not report themselves failing to provide adequate care as this can be a moral issue. In many settings, because critical PPE components were in insufficient supply during the COVID-19 outbreak, many healthcare personnel worldwide hesitated to offer patient care [49].

Most nurses and midwives reuse the PPEs, including the non-reusable ones, which is inappropriate. Reusing was connected to the absence of PPEs in the current study. Similarly, a study undertaken among the adult population in Hong Kong revealed that 99% of the adult population reused masks due to limited supply and uncertainty about their availability in the future [49]. However, this was a different study population compared to the present study, although the reasons for reuse remain similar in both settings.

**Study limitations:** the study could have yielded better results had it been conducted at a site specifically for nursing COVID-19 patients. Since this was a once-off and quantitative study, there was no further information to learn about the inner feelings of the healthcare providers, which would have given us an insight into the magnitude of the problem of inadequate PPEs. Health centres could not be accessed to verify information given by participants, so there was a likelihood of an under or overestimation.

## Conclusion

The study found that most nurses and midwives did not have access to adequate PPEs but reported that they had enough surgical masks, followed by gloves and long-sleeved gowns, and the least available were eye goggles. About three-quarters of study participants said they lacked sufficient PPEs in total. Although the problem of inadequate PPEs is not only peculiar to Zambia, this inadequacy has adverse effects on the quality of health care provided during a pandemic such as COVID-19 because it might contribute to healthcare providers' reluctance to offer quality care. Due to the virulence of the virus, many healthcare workers have lost their lives after contracting COVID-19. The situation might even worsen in remote areas where most medical supplies cannot reach the facilities. Nurses and midwives rendering care in maternity settings still have to nurse the patients as usual and remain at risk of contracting the disease.

### What is known about this topic

- *COVID-19 is a significant health problem globally;*
- *PPEs are an essential component required for the prevention of COVID-19 in health care providers;*
- *Many countries, including developed ones, run out of PPES, thus putting the health care providers at risk of contracting COVID-19*.

### What this study adds

- *There is a paucity of data concerning PPEs in maternity wards;*
- *Due to their propensity for taking time with each patient, midwives are more likely to contract COVID-19 while caring for patients in maternity wards;*
- *In this study, we found that reusing PPEs was associated with the availability of PPEs in health facilities*.
